# 4405 Chronic Disease in Indiana – Using a Community Health Matrix to Determine Health Factors for Indiana Counties

**DOI:** 10.1017/cts.2020.263

**Published:** 2020-07-29

**Authors:** Sarah Wiehe, Aaron Zych, Karen Hinshaw, Ann Alley, Gina Claxton, Dennis Savaiano

**Affiliations:** 1 Indiana University School of Medicine; 2 Indiana University; 3 Indiana State Department of Health; 4 Community Health Partnerships; 5 Purdue University

## Abstract

OBJECTIVES/GOALS: The goal of this project was to inform four chronic disease initiatives, working together on the team Connections IN Health, and counties in Indiana on certain areas of need to assist them in collaborative planning. The chronic diseases focused on include diabetes, cardiovascular disease, stroke, asthma, lung cancer and obesity. METHODS/STUDY POPULATION: Chronic disease health outcomes and social determinants of health indicators were identified in all 92 Indiana counties. Counties were compared by composite z scores in a matrix to determine the 23 counties with the poorest health statistics for diabetes, cardiovascular disease, stroke, asthma, lung cancer, obesity and life expectancy. Qualitative data were used to identify local health coalitions that have the capacity and desire to work with Connections IN Health to improve these health outcomes. With input from partners, the counties were narrowed to 10 that were identified as those with the most need in the specific areas of chronic disease that the initiatives focus on. The team will begin listening sessions with two of these counties to identify strategic partnerships, funding sources, and evidence-based programs to address community-identified health priorities. RESULTS/ANTICIPATED RESULTS: The 23 counties with the poorest health outcomes related to chronic disease and factors were Blackford, Clark, Clay, Fayette, Fulton, Grant, Greene, Howard, Jay, Jennings, Knox, Lake, LaPorte, Madison, Marion, Pike, Scott, Starke, Sullivan, Vanderburgh, Vermillion, Vigo, and Washington. There was significant overlap in low z score rankings for individual health and social determinants of health measures among these 23 counties. The following 10 counties were selected for focus in the next five years based on partner input: Blackford, Clay, Grant, Jennings, Lake, Madison, Marion, Starke, Vermillion, and Washington. The Connections IN Health team has initiated listening sessions in Grant and Vermillion Counties (with data for presentation at the ACTS meeting). DISCUSSION/SIGNIFICANCE OF IMPACT: This mixed methods approach using existing data and partner input on county capacity/readiness directed Connections IN Health to counties with the most need for coalition efforts. Engagement within each county will inform next steps (e.g., capacity building, partnership development, applications for funding, implementation of evidence-based programs) and specific health focus area(s).

